# Surveilling microglia dampens neuronal activity: operation of a purinergically mediated negative feedback mechanism

**DOI:** 10.1038/s41392-021-00586-4

**Published:** 2021-04-17

**Authors:** Peter Illes, Alexei Verkhratsky, Yong Tang

**Affiliations:** 1 Rudolf Boehm Institute for Pharmacology and Toxicology, University of Leipzig, Leipzig, Germany; 2 School of Acupuncture and Tuina, Chengdu University of Traditional Chinese Medicine, Chengdu, China; 3 International Collaborative Center on Big Science Plan for Purinergic Signaling, Chengdu University of Traditional Chinese Medicine, Chengdu, China; 4 Faculty of Biology, Medicine and Health, The University of Manchester, Manchester, UK

**Keywords:** Neuroimmunology, Cellular neuroscience

The recent paper published in *Nature* by Badimon et al. demonstrates that neuronal activity via the stimulation of surveilling microglia feeds back to inhibit neuronal networks.^1^ This effect is mediated by the release of ATP from the terminals of excitatory neurons; subsequently, ATP is degraded to adenosine by microglial enzymes, imposing an inhibitory, A1 receptor (R)-mediated control on the hyperactive neurons themselves.

**Fig. 1:**
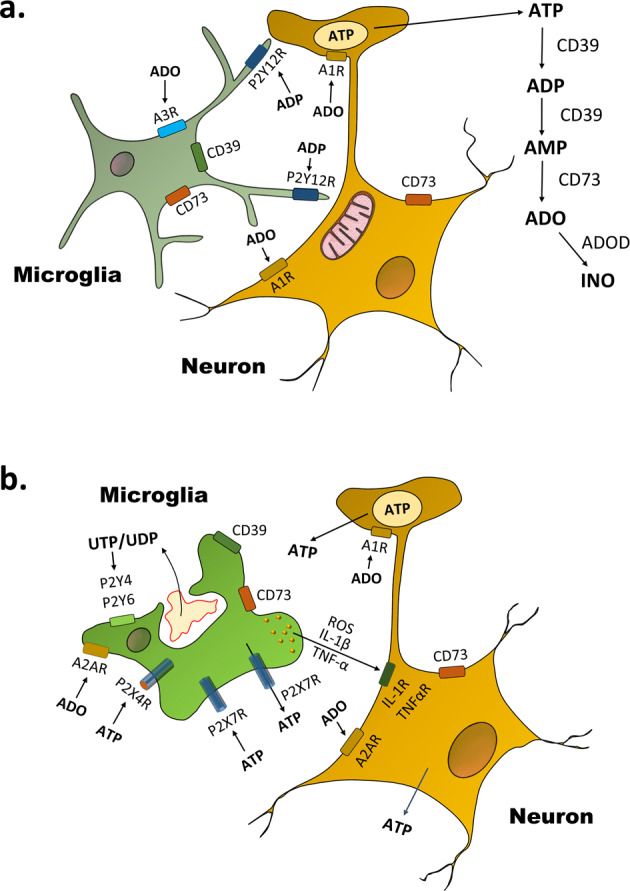
Microglia–neuron cross-talk at different microglial activation states. **a** Surveilling/ramified microglia send their processes to the ATP-releasing terminals of neurons as well as to their somata where the plasma membrane covers ATP-producing mitochondria. Neuronally released ATP is gradually degraded by CD39 to ADP and AMP, and then by CD73 to adenosine (ADO). Adenosine is inactivated by adenosine deaminase (ADOD) to inosine (INO). P2Y12Rs respond to transmitter ATP or somatically released ATP by increased coverage by microglial processes of these neuronal sites. A3Rs and P2Y12Rs act in concert to promote microglial process motility. Adenosine feeds back via presynaptic or somatic A1Rs to inhibit neuronal activity. **b** Activated/ameboid microglia phagocytose bacteria or cellular debris. ATP may be again released as a neurotransmitter but may also leave the neuronal cytoplasm via holes in the damaged cellular membrane. In addition, ATP is outpouring from neighboring astrocytes mainly through connexin channels and pannexin-1 hemichannels in a nonexocytotic manner. Instead of P2Y12Rs, now P2X7Rs are the main microglial sites at which ATP acts. P2X7Rs are pathways for the release of ATP, and more importantly, they induce the production/release of proinflammatory cytokines (IL-1β, TNF-α) and reactive oxygen species (ROS). These cell products cause neuroinflammation and subsequent neuronal damage through the activation of their specific receptors (IL-1R, TNFαR). P2X7Rs act in synchrony with P2X4Rs. A2ARs promote the transition of ramified microglia to ameboid microglia. UDP-sensitive P2Y6Rs trigger microglial phagocytosis, while UTP (ATP)-sensitive P2Y4Rs trigger microglial pinocytosis. UTP/UDP derives from the nucleus of injured cellular material. Whereas A1Rs at neurons are inhibitory, A2ARs are excitatory and they promote neurodegeneration

Microglia are the resident macrophages of the CNS.^2^ The microglial population is highly static and maintained during adulthood. For many years, it was assumed that microglia exist in an inactive, ramified state (Fig. 1a) and an activated, ameboid state (Fig. 1b), the latter one being responsible for the typical microglial reactions such as phagocytosis and the release of bioactive molecules (proinflammatory cytokines, chemokines, proteases, reactive oxygen species, and probably also the excitotoxic ATP/glutamate). Ameboid microglia possesses two ionotropic ATP-sensitive receptors (P2X4, P2X7) and two G-protein-coupled nucleotide-sensitive receptors (P2Y4, P2Y6). In addition, microglial cells are endowed with the G-protein-coupled adenosine-sensitive receptors A2A and A3.

In spite of the early, rigid distinction into inactive/ramified and activated/ameboid forms of microglia, more recently, in vivo two-photon imaging in the neocortex demonstrated that microglial cells are highly active in their presumed resting state, continuously surveying their microenvironment with extremely motile processes and protrusions.^3^ The motility of microglia was steered by their P2Y12Rs, stimulated by ATP, released from any type of damaged CNS constituent or alternatively from nerve terminals utilizing ATP as a (co)transmitter substance. It has been shown that neurons signal to microglial processes by ATP, noradrenaline, or a chemokine (fractalkine, CX3CL1) to stimulate P2Y12Rs, β_2_ adrenoceptors, and CX3CR1, respectively. In this respect, it is important to note that astrocytes, which form a functional unit with neighboring neurons, may also release ATP both by exocytosis and by diffusion through connexin hemichannels/pannexin channels.

Roles are now recognized for microglia in early CNS development where they clear apoptotic cells, facilitate brain wiring, and regulate synaptic development. In adults, microglia participate in mechanisms underlying learning and behavior, e.g., by the shaping of neuronal circuits and the release of brain-derived neurotrophic factor (BDNF). This BDNF has been shown to modify synaptic plasticity by increasing neuronal tropomyosin-related kinase receptor B phosphorylation.

The ATP-induced signaling to microglia has been elucidated in detail in the past years and has been shown to, e.g., regulate seizure activity in consequence of synchronous firing of many glutamatergic neurons.^4^ The released glutamate was suggested to activate neuronal NMDA-Rs, triggering Ca^2+^ influx into the intracellular space, subsequent ATP release, and microglial response through P2Y12Rs. P2Y12R KO mice exhibited reduced seizure-induced increases in microglial process numbers and worsened seizure behavior. Although microglia may interact with neurons at synaptic sites, microglia–neuron junction may be formed also at the somatic plasma membrane covering clusters of the mitochondria.^5^ Microglia at somatic junctions appear to sense changes in the metabolic state of neurons through the local exocytosis of ATP produced by the mitochondria. Intracerebral application of a P2Y12R inhibitor prevented mitochondrial fragmentation during experimental stroke, by precluding stroke-induced increases of microglial process coverage of somatic junctions.

Although neuronal signaling to microglia has been repeatedly identified, there appeared to be no evidence hitherto of a reversely directed signaling mechanism from microglia toward neurons. However, microglia apparently respond to neuronal activation by exerting a feedback break on neurons, and ablation of microglia amplifies and synchronizes the activity of neurons, leading to seizures^1^ (see also above). Microglia respond to neuronal activation by distinct changes in their gene expression profile by upregulating genes involved in chemotaxis and actin filament polymerization necessary for microglial process motility. In parallel, brain-wide ablation of microglia by genetic deletion of the prosurvival receptor colony-stimulating factor 1 receptor (CSF1R) rendered mice hyperresponsive to seizure induction by otherwise subthreshold doses of the convulsant drugs kainic acid or picrotoxin. Similarly, pharmacological stimulation of dopamine D1R-expressing striatal neurons by the D1R agonistic SKF81297 caused increases in motor activity and seizures in mice. The specific deletion of the *IL-34* gene in neural progenitor cells leading to deficiency in interleukin-34 (IL-34), which activates CSF1R signaling, resulted in the loss of microglia in the gray matter but not in the white matter of the striatum. In consequence, mice with gray matter-specific ablation of microglia responded to D1R agonistic treatment with the induction of seizures, whereas mice with white matter-specific ablation did not.

Two-photon microscopy coupled with [Ca^2+^]_i_ measurement in the in vivo striatum showed that ablation of microglia led to increased striatal neuron synchrony. This is likely to contribute to seizures in microglia-deficient mice. Furthermore, using two-photon microscopy of enhanced green fluorescence (EGFP)-expressing microglia in the cortex demonstrated that the activation of neurons resulted in the decrease of microglial motility manifested as a retarded extension and retraction of microglial processes.

ATP is known to be enzymatically degraded by a cascade of enzymes, first to ADP and AMP (CD39, ectonucleoside triphosphate diphosphohydrolase 1, NTPDase1) and then from AMP to adenosine (CD73, 5’-nucleotidase). Eventually, adenosine is inactivated by adenosine deaminase. CD39 is primarily expressed in microglia, while CD73 is also expressed in neurons. This suggests that microglia can contribute to the production of adenosine alone or by involving neighboring neurons. Microglia-specific deletion of *Entpd1* (which encodes CD39) resulted in a higher susceptibility to D1R agonist-induced seizures. In addition, pharmacological inhibition of A1R activity or D1 neuron-specific ablation of A1R expression in mice triggered an exaggerated D1 neuron response that recapitulated the effect of microglia ablation.

It was concluded that neuronal ATP increases the duration of the microglia–neuron contact via P2Y12R stimulation and in parallel leads to enhanced enzymatic degradation of this ATP to adenosine. Adenosine thereafter suppresses neuronal activity by stimulating neuronal A1Rs with obligatory behavioral consequences. Hence, surveilling microglia feed back to neurons is necessary in order to limit excessive activity, e.g., during epileptic seizures. Although the highlighted article deals only with a massive increase in neuronal activity during epileptic seizures, a continuous, although the less intensive promotion of ATP release from neurons/non-neuronal elements may occur also during various degenerative processes of the CNS (Alzheimer’s disease, Parkinson’s disease, multiple sclerosis, and amyotrophic lateral sclerosis). This ATP may stimulate microglial P2Y12Rs and in consequence exert a feedback inhibitory control of neuronal activity via A1R occupation and limitation of neuronal function. Elucidation of these mechanisms may have a broader impact on research and fertilize future experimental work.
